# Supplementary material for: A kinase-deficient NTRK2 splice variant predominates in glioma and amplifies several oncogenic signaling pathways
Source: Nat Commun. 2020 Jun 12;11:2977. doi: 10.1038/s41467-020-16786-5 (PMC7293284; doi:10.1038/s41467-020-16786-5)
Supplement: Supplementary file 1 — Supplementary Information [file 41467_2020_16786_MOESM1_ESM.pdf]

A kinase-deficient *NTRK2* splice variant predominates in glioma and amplifies several oncogenic signaling pathways

Pattwell et al.

Supplementary Figures 1-9
Supplementary Table 1

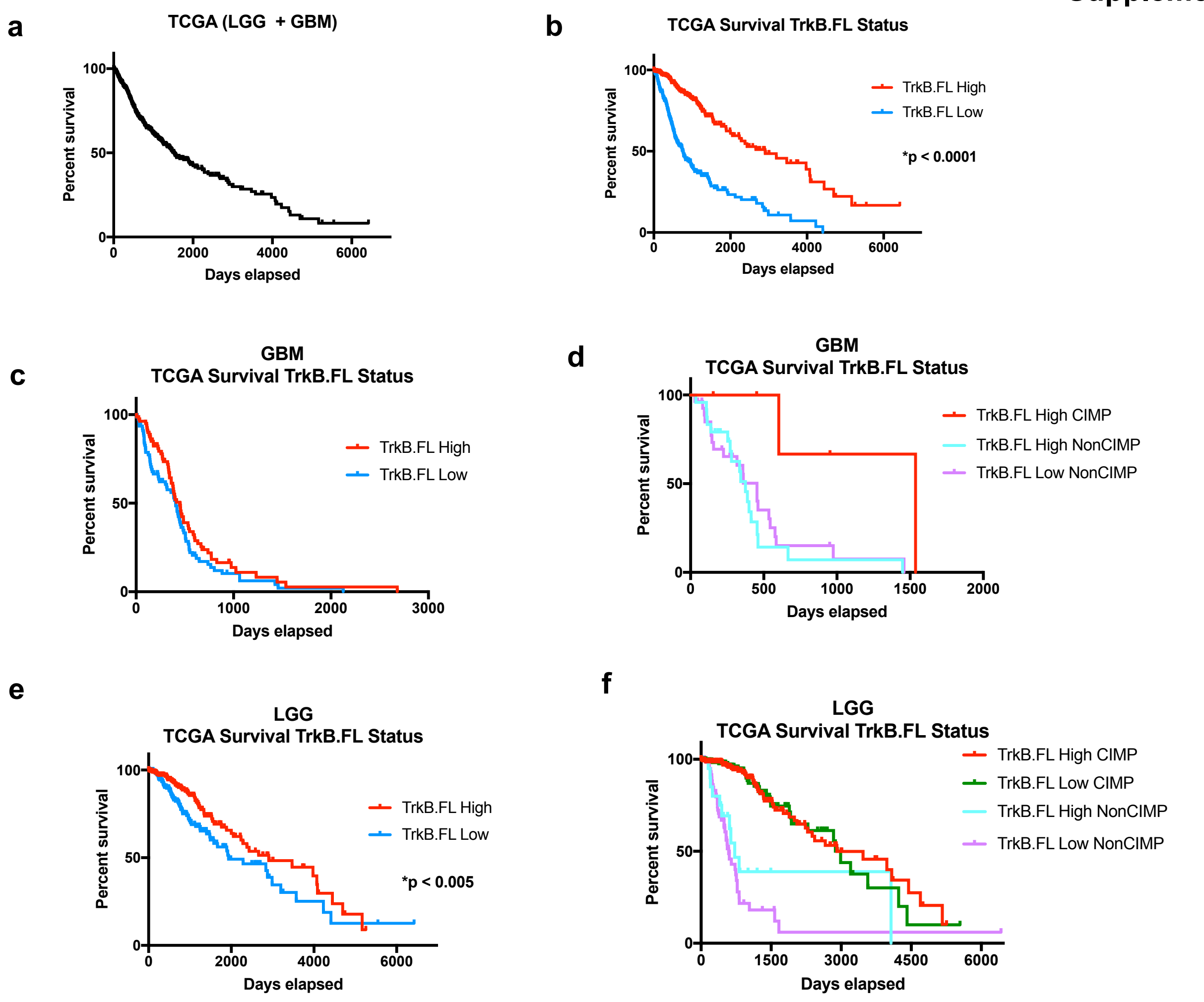

Supplementary Fig. 1: Survival curves from The Cancer Genome Atlas (TCGA). Survival curve for all TCGA low grade glioma (LGG) and glioblastoma multiforme (GBM) samples combined **(a)** compared to survival curve for all combined TCGA samples divided by TrkB.FL status **(b)** shows that higher TrkB.FL expression does *not* correlate with worse survival and correlates with *longer* survival (above median = TrkB.FL high; below median = TrkB.FL low; 2907 days vs 758 days (log rank hazard ratio 0.2827, 95% confidence interval 0.2195-0.3641, $p<0.0001$)). When stratified by tumor type, there is a trend for TrkB.FL status to correlate with longer survival in GBM (448 days vs 408 days) but this is not statistically significant **(c,d)**. There is a significant effect of TrkB.FL status on survival in LGG where high TrkB.FL corresponds with longer survival **(e, f)** 2907 days vs 1933 days (log rank hazard ratio 0.6281, 95% confidence interval 0.4411-0.8945, $p<0.005$).

Supplementary Fig. 2

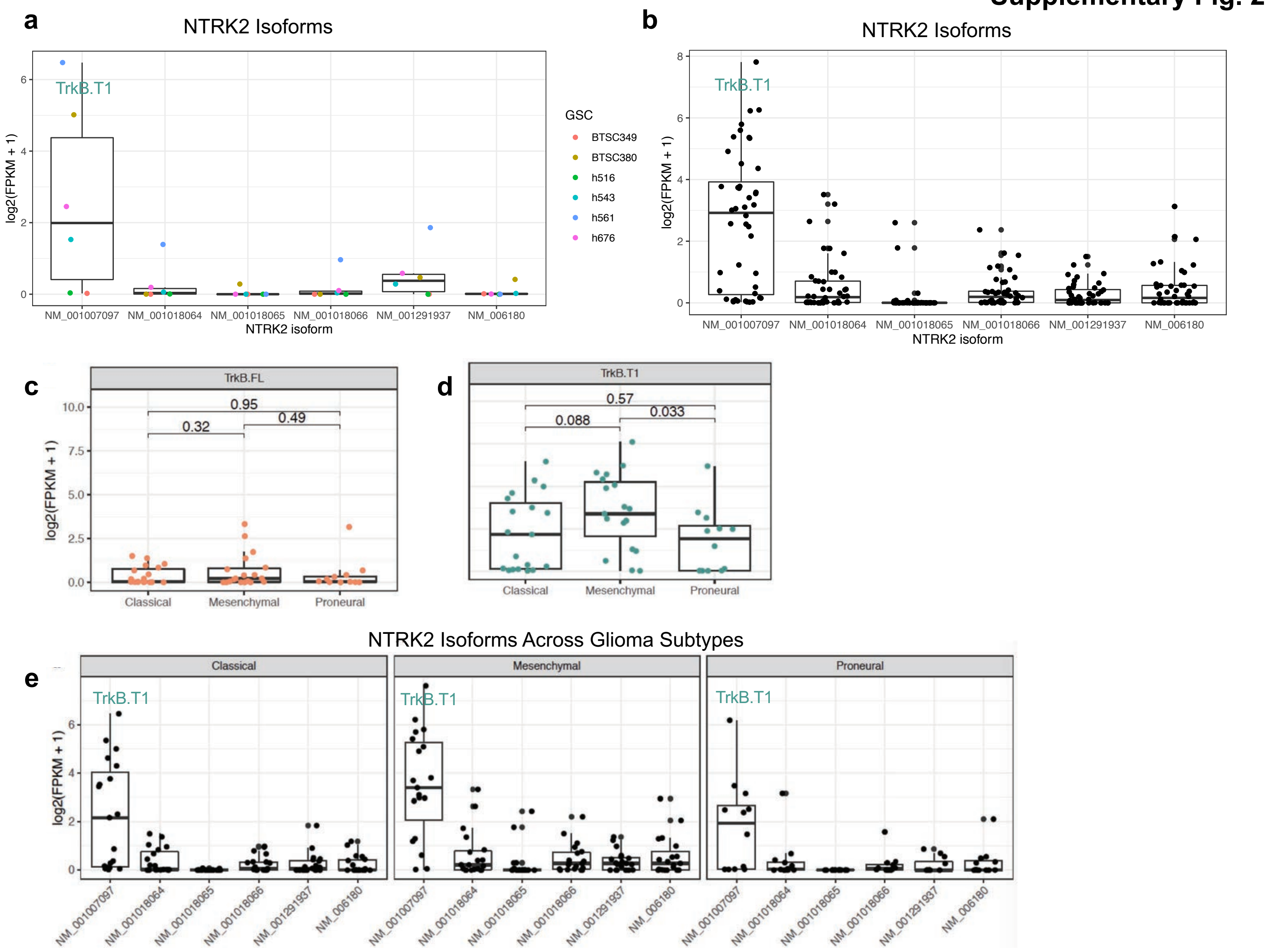

Supplementary Fig. 2: NTRK2 Transcript Analysis shows TrkB.T1 to be the most predominant NTRK2 isoform across a large sample of human glioblastoma stem cell (GSC) lines. TrkB.T1 is the predominant NTRK2 isoform expressed in 6 in house GSCs lines (BTSC349, BTSC349, h543, h516, h561 and h676) **(a)** and in 44 additional GSC lines derived from primary tumors[1] **(b)**. TrkB.FL expression remains low across all GSC subtypes while TrkB.T1 expression is increased compared to other NTRK2 variants across GSC subtypes (50 total GSC lines) **(c, d, e)**. Data shown in **(c)** contains p-values for t-tests, two-sided. Data are represented as boxplots **(a - e)**, where the middle line is the median, the lower and upper hinges correspond to the first and third quartiles (the 25th and 75th percentiles), the upper whisker extends from the hinge to the largest value no further than 1.5 * IQR from the hinge (where IQR is the inter-quartile range, or distance between the first and third quartiles) and the lower whisker extends from the hinge to the smallest value at most 1.5 * IQR of the hinge while data beyond the end of the whiskers are outlying points that are plotted individually [2].

Supplementary Fig. 3

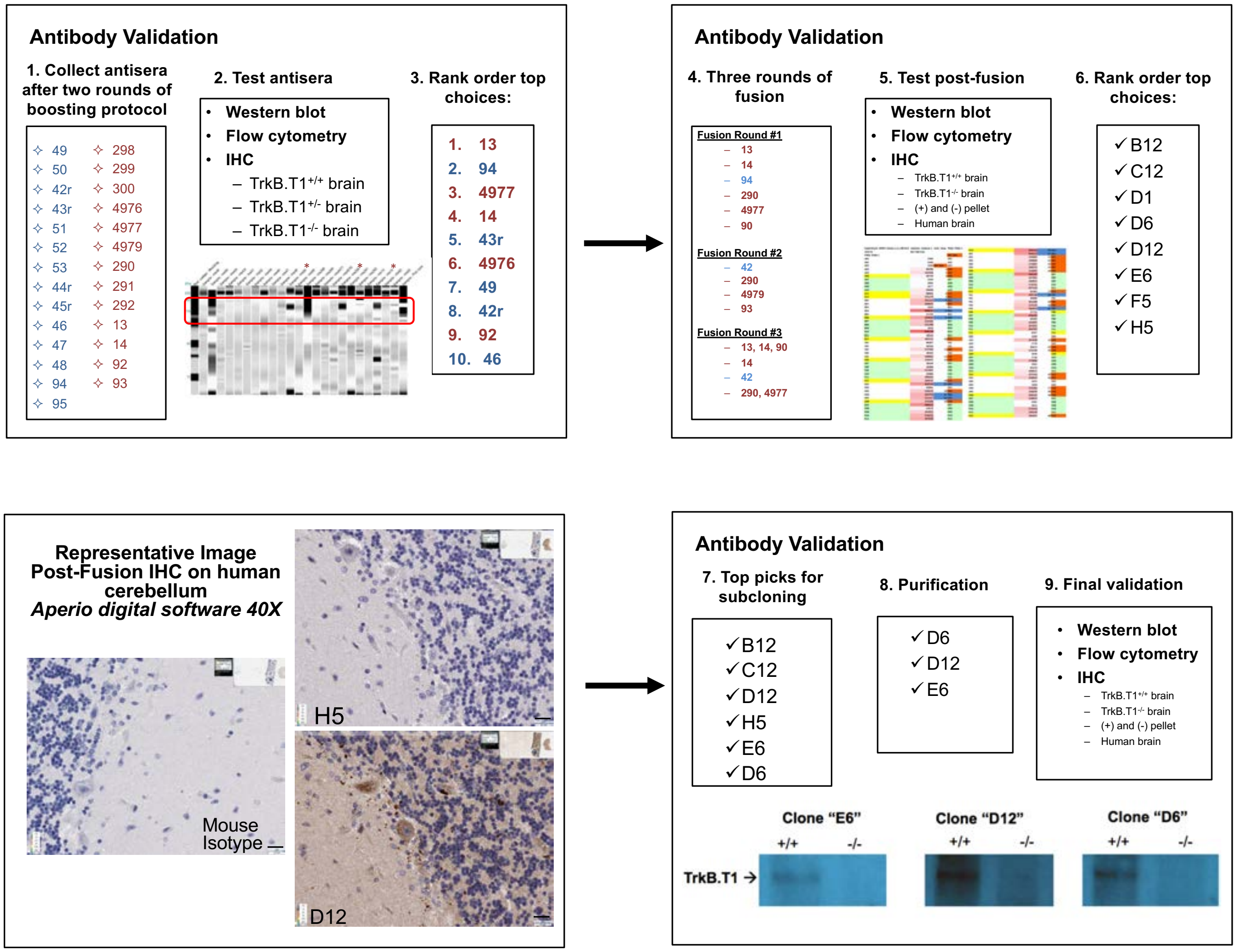

Supplementary Fig. 3: Summary of antibody validation process. Mice used for TrkB.T1-specific antibody development included standard CD1, BALBC, A/J mice (mouse IDs shown in blue) and TrkB.T1$^{-/-}$ mice (mouse IDs shown in red). Prior to fusion, antisera from each mouse was tested for positive staining in immunohistochemistry on TrkB.T1$^{+/+}$ wild-type mouse brain, TrkB.T1$^{+/-}$ mouse brain, and TrkB.T1$^{-/-}$ mouse brain and normal human brain. Clone selection protocol and representative image of positive western blots during antibody development using WES system. WES blots were confirmed with western blotting using Life Tech XCell SureLock™ Mini-Cell electrophoresis system using wild-type mouse brain and TrkB.T1$^{-/-}$ brain. Western blots were performed in duplicate using technical replicates of each clone on biologically independent samples, and representative images were chosen, TrkB.T1 band at 95 kDa. Aperio 40X scale bars = 25 μm.

Supplementary Fig. 4

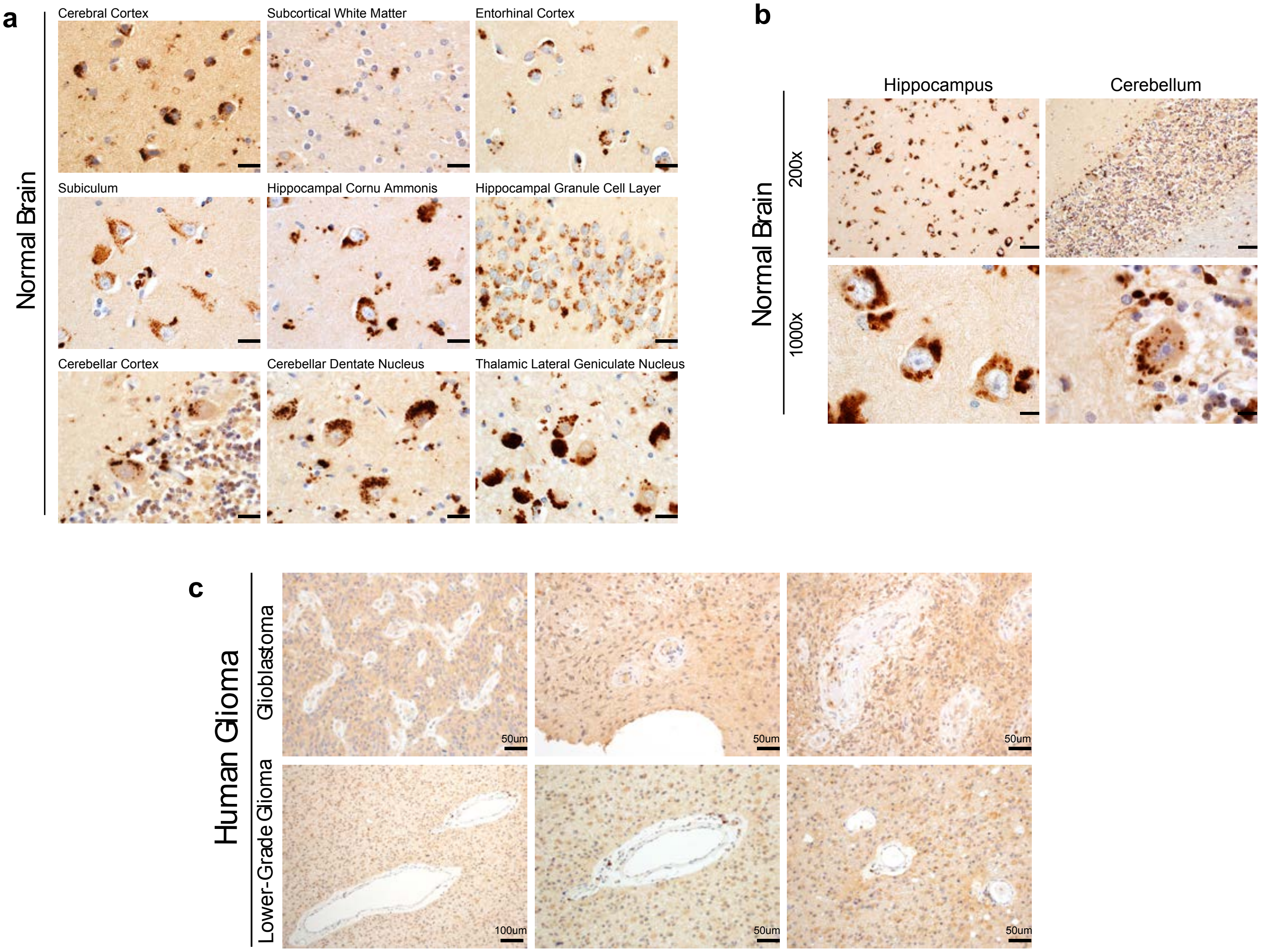

Supplementary Fig. 4: TrkB.T1 immunostaining in normal human brain and glioma. (a) Immunohistochemical staining demonstrating regional distribution of TrkB.T1 in normal human brain regions with punctate, vesicular pattern. Photomicrographs are at 400-600X. **(b)** Regional distribution of TrkB.T1 in normal hippocampus and cerebellum shows punctate, vesicular pattern at higher magnification. Photomicrographs are as specified at both 200X (scale bar = 50 μm) and 1000X (scale bar = 10 μm) to highlight punctate, vesicular pattern. **(c)** TrkB.T1 immunostaining of GBM and diffuse gliomas shows diffuse positive stain throughout tumor and negative staining in blood vessels. Photomicrographs are at 10-20X (scale bars individually labeled). Immunohistochemistry was performed on independent biological samples of each brain region and tumor type, in replicates of 3-5. Representative images were chosen and additional images are shown in Fig.4.

Supplementary Fig. 5

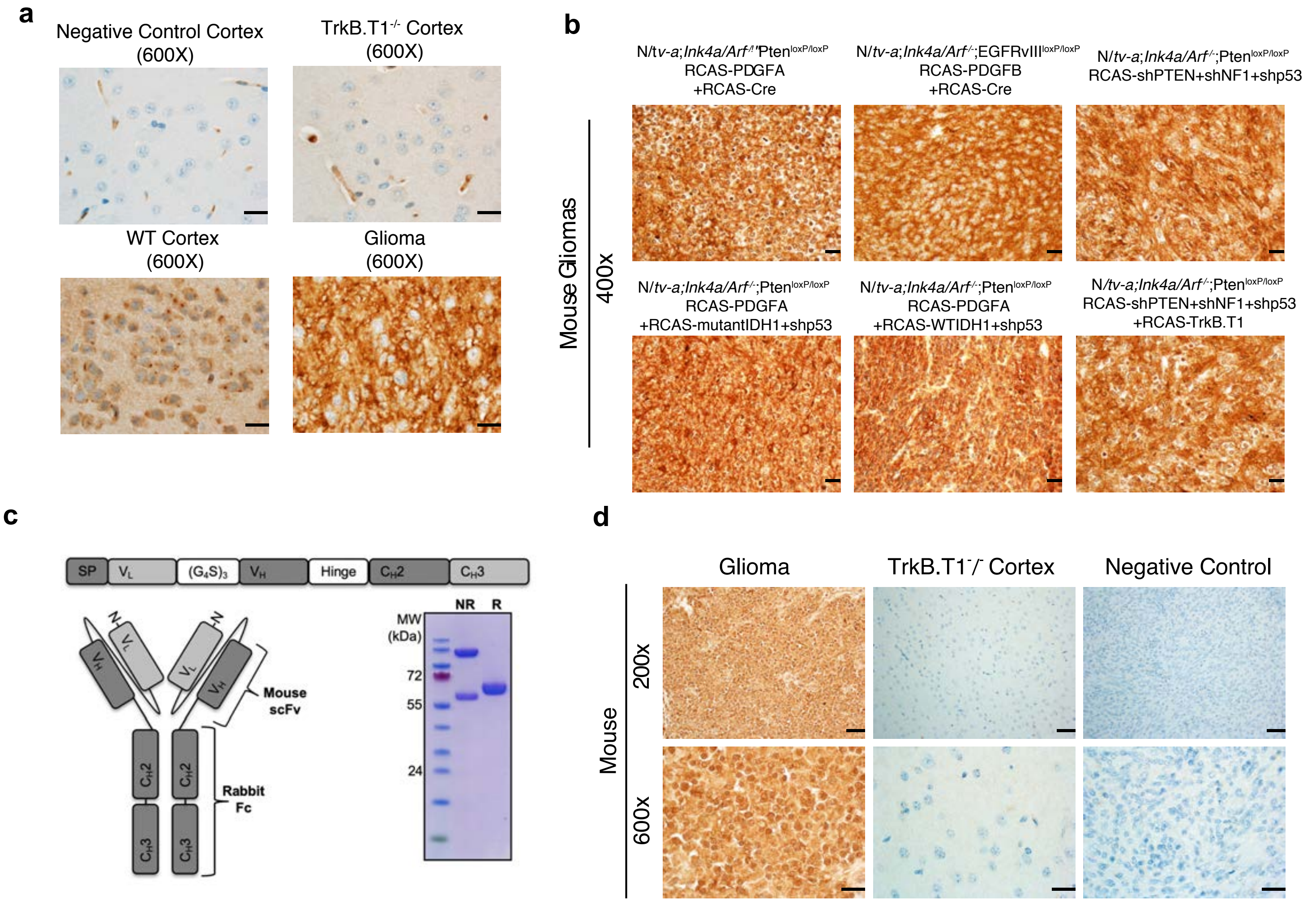

Supplementary Fig. 5: TrkB.T1 immunostaining in mouse gliomas and TrkB.T1 recombinant rabbit antibody. **(a)** TrkB.T1 immunostaining of normal mouse cortex shows comparable staining in the negative (no primary antibody) control and TrkB.T1$^{-/-}$ cortex, punctate/vesicular staining normal wild-type (WT) mouse cortex and strong/diffuse staining in tumor. Immunostaining of various RCAS-driven mouse gliomas shows positive staining in tumor regardless of genetic background or RCAS-injection combination **(b).** Schematic showing the domain architecture of the SPEH1_D12 scFv-Fc fusion protein and sodium dodecyl sulfate polyacrylamide gel showing the purified recombinant protein expressed in mammalian cells under non-reducing and reducing conditions **(c)**. TrkB.T1 immunostaining in mouse gliomas using recombinant TrkB.T1 fusion protein with rabbit secondary antibodies recapitulates staining patterns using TrkB.T1 mouse antibody, shown in **(a)** (and **Fig. 4** and **Fig. Supplementary Fig. 4)**, showing diffuse staining in mouse tumor with a lack of staining in rodent TrkB.T1$^{-/-}$ cortex and negative control (no primary antibody) **(d)**. Photomicrographs are as specified at 200X (scale bar = 50 μm), 400X (scale bar = 20 μm), 600X (scale bar = 20 μm). Immunohistochemistry was performed on independent biological samples of each brain region and tumor type, in replicates of 3-5. Representative images were chosen and additional images are shown in Fig.4.

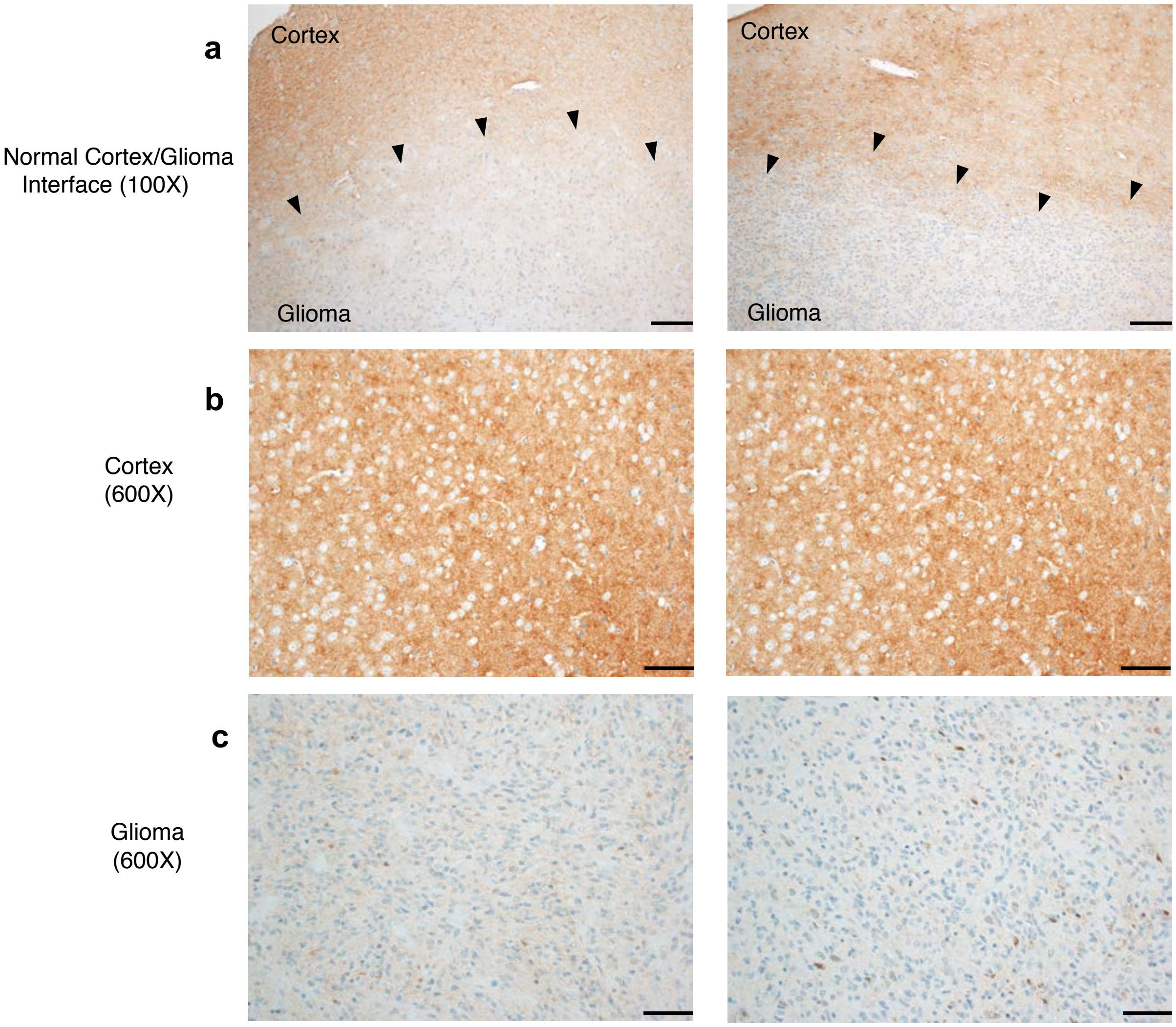

Supplementary Fig. 6: Immunostaining of TrkB.FL. Immunostaining with an antibody designed against the TrkB kinase (which therefore detects TrkB.FL, but not TrkB.T1), shows contrasting patterns to TrkB.T1 staining shown in **Fig. 4** and **Supplementary Fig. 4, Supplementary Fig. 5.** TrkB.FL staining is strong in the normal cortex and virtually absent in tumor **(a,b,c)**. Photomicrographs are as specified at 100X (scale bar = 100 μm) and 600X (scale bar = 20 μm). Immunohistochemistry was performed on independent biological samples of each normal brain/tumor interface, in replicates of 3-5.

Supplementary Fig. 7

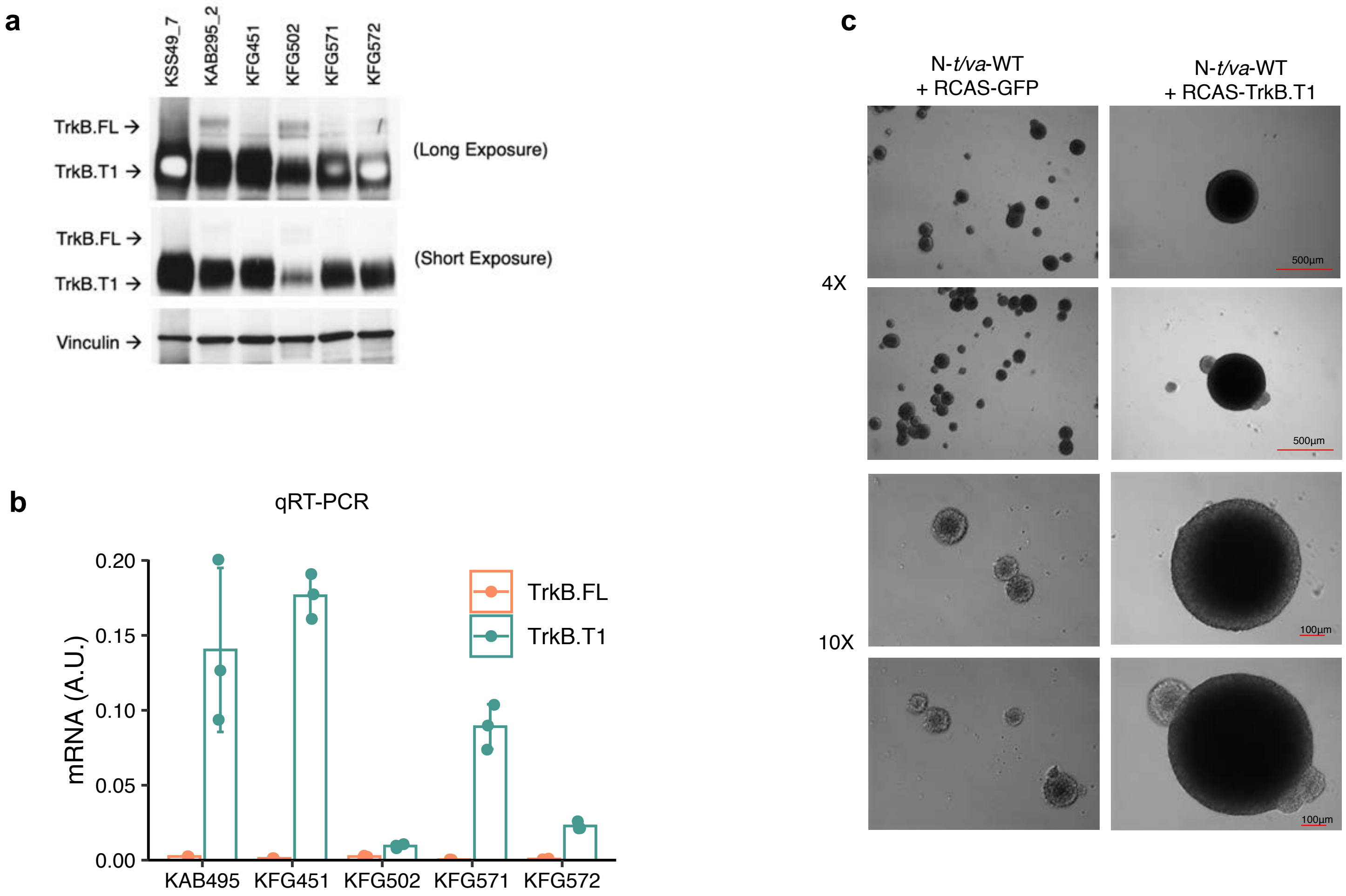

Supplementary Fig. 7: Mouse tumorspheres show increased TrkB.T1 expression and RCAS-TrkB.T1 enhances nestin/*tv-a* primary mouse neurosphere diameter. (a) Mouse tumorsphere lines show a predominance of TrkB.T1 (95 kDa) compared to TrkB.FL (145 kDa) via western blot (KSS49_7: wildtype; KAB295_2: wildtype; KFG451: PDGFB + sgRNA PTEN; KFG502: PDGFB + sgRNA p53; KFG571: PDGFB + sgRNA CDKN2a; KFG572: PDGFB + sgRNA CDKN2a) and **(b)** qRT-PCR (cell lines analyzed in triplicate; n = 3 per cell type; error bars represent standard deviation). Western blotting was performed on triplicate samples and representative images were chosen with vinculin loading control (124 kDa). qRT-PCR bars for TrkB.T1 shown in teal, qRT-PCR bars TrkB.FL shown in orange. **(c)** Representative images of neural stem cells isolated from subventricular zone of nestin/*tv-a* wild-type postnatal day 1 (P1) pups were infected with RCAS-GFP or RCAS-TrkB.T1 and allowed to form neurospheres. Images taken at 4X (scale bar = 500 *µ*m) and 10X (scale bar = 100 *µ*m), as indicated, demonstrate enhanced neurosphere diameter in RCAS-TrkB.T1 spheres, consistent with previous findings [3].

Supplementary Fig. 8

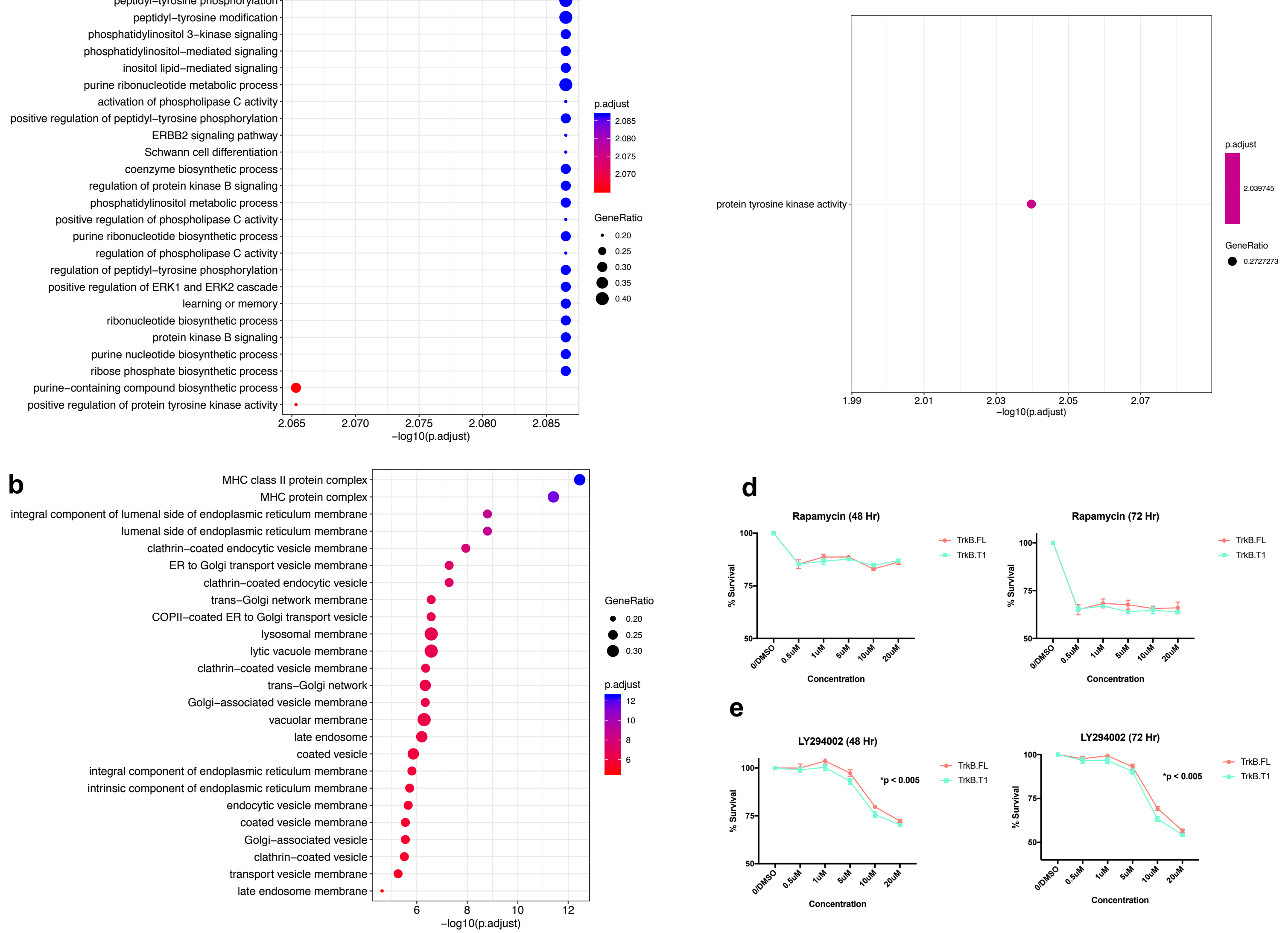

Supplementary Fig. 8: TrkB.T1 upregulates genes involved in PI3K/Akt signaling and downregulates genes associated with clathrin coated vesicles and endosomes in human neural stem cells (NSCs) and TrkB.T1 NSCs are more sensitive to PI3K inhibitor LY294002. (a) Biological process GO terms for genes upregulated in TrkB.T1 transduced NSCs compared to TrkB.FL transduced NSCs revealed genes involved in PI3K/Akt pathway, ERBB2, and endosomal compartments. **(b)** Cellular compartment GO terms revealed a host of downregulated genes in clathrin-coated vesicles and endosomes while **(c)** molecular process terms showed upregulated protein kinase activation genes in TrkB.T1 transduced NSCs compared to TrkB.FL-transduced NSCs. Treatment with rapamycin yielded similar dose-dependent results in both TrkB.T1 and TrkB.FL transduced NSCs at both 48 and 72 hours **(d)**, while TrkB.T1 transduced NSCS were significantly more sensitive to LY294002 than TrkB.FL transduced NSCs in a dose dependent manner at 48 hours ($F(1,24) = 10.03$; p=0.0042) and at 72 hours ($F(1,24) = 12.91$; p=0.0015) **(e)**. For rapamycin drug studies, n = 3 plates of TrkB.T1-transduced NSCs, 3 plates of TrkB.FL-transduced cells, for each time point (3 plates of each condition for 48 hrs, 3 plates of each condition for 72 hrs). For LY294002 drug studies, n = 3 plates of TrkB.T1-transduced NSCs, 3 plates of TrkB.FL-transduced cells, for each time point (3 plates of each condition for 48 hrs, 3 plates of each condition for 72 hrs). TrkB.T1-transduced NSCs shown in teal, TrkB.FL-transduced NSCs shown in orange.

Supplementary Fig. 9

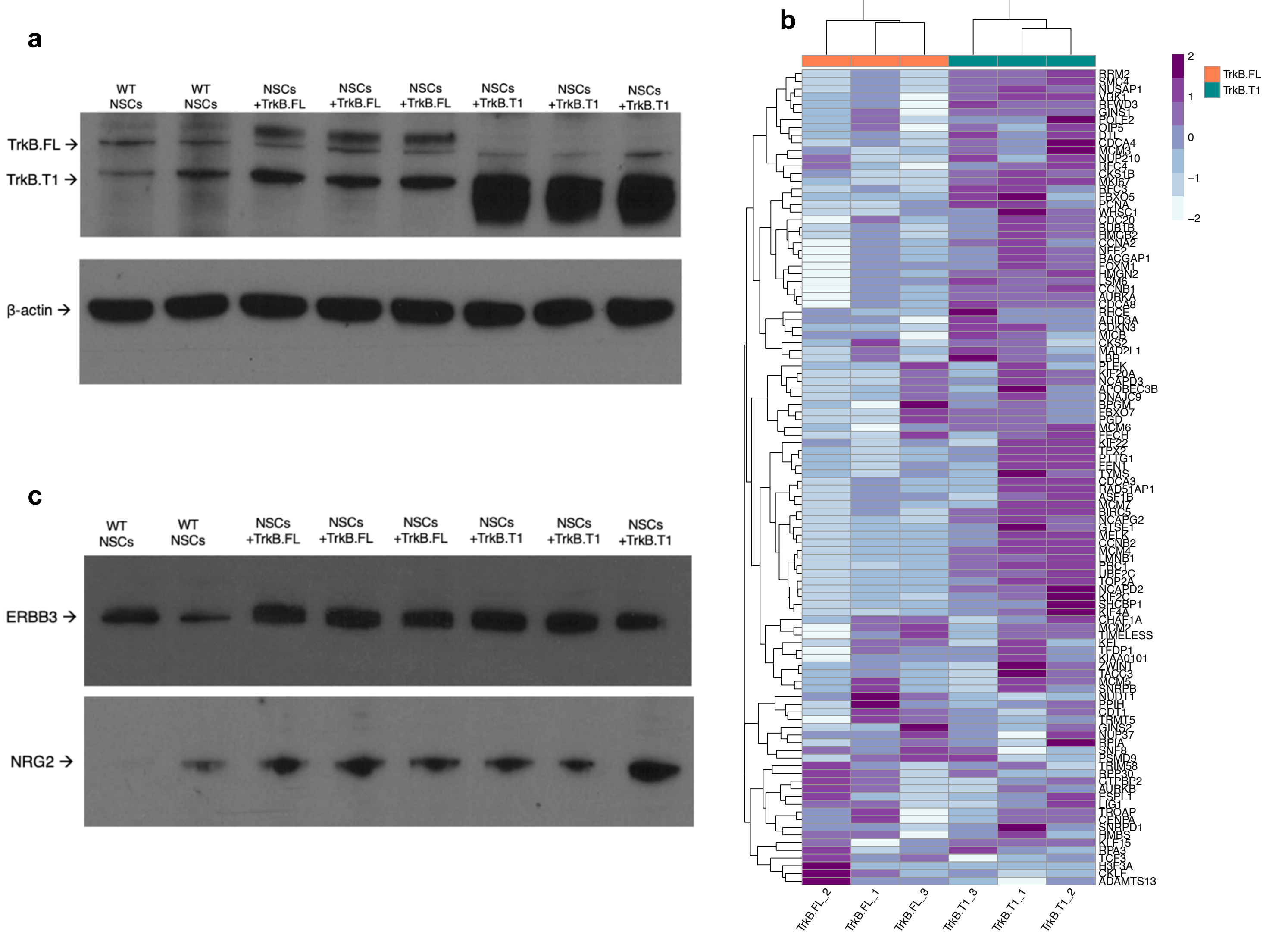

Supplementary Fig. 9: NSC RNA Seq validation by protein analysis. (a) Western blots for TrkB show that (corresponding to RNA Seq data in **Fig. 5**. and **Supplementary Datasheet 6**), TrkB.FL protein (145 kDa) levels are increased in pLJM1-TrkB.FL transduced NSCs while TrkB.T1 protein (95 kDa) levels are increased in pLJM1-TrkB.T1 transduced NSCs, compared to the endogenous expression of either isoform in NSCs. **(b)** TrkB.T1 transduced NSCs show increases in genesets previously characterized as makers of a cell's proliferative index [4,5]. **(c)** Western blot shows increased expression of ERBB3 (185 kDa) and ligand NRG2 (92 kDa) protein compared to endogenous NSC expression for these proteins. TrkB.T1-transduced NSCs shown in teal, TrkB.FL-transduced NSCs shown in orange. Western blotting was performed in duplicate and triplicate, on biologically independent samples, and representative images were chosen.

Supplementary References

1. Mack, S.C. *et al.* Chromatin landscapes reveal developmentally encoded transcriptional states that define human glioblastoma. *J Exp Med* **216**, 1071-1090 (2019).
2. Hadley, W. *Ggplot2*, pages cm (Springer Science+Business Media, LLC, New York, NY, 2016).
3. Tervonen, T.A. *et al.* Overexpression of a truncated TrkB isoform increases the proliferation of neural progenitors. *Eur J Neurosci* **24**, 1277-85 (2006).
4. Ramaker, R.C. *et al.* RNA sequencing-based cell proliferation analysis across 19 cancers identifies a subset of proliferation-informative cancers with a common survival signature. *Oncotarget* **8**, 38668-38681 (2017).
5. Venet, D., Dumont, J.E. & Detours, V. Most random gene expression signatures are significantly associated with breast cancer outcome. *PLoS Comput Biol* **7**, e1002240 (2011).

Supplementary Table 1. Commercially available TrkB antibodies.

Commercial vendor	Catalog #	Host Species	Reactivity	Immunogen	Suggested Application
Abcam	ab33655	Rabbit	Mouse, Rat, Human	The entire extracellular domain (corresponding to residues 1 to 429) of rat TrkB.	WB, IP, ICC/IF, IHC-P
Abcam	ab18987	Rabbit	Mouse, Rat, Chicken, Human	Synthetic peptide corresponding to Human TrkB. Surrounding amino acid 810 of human TrkB. (Peptide available as ab52216)	WB, IP, ICC/IF, IHC-P
Abcam	ab134155	Rabbit monoclonal [EPR1294] TrkB	Human	Synthetic peptide (the amino acid sequence is considered to be commercially sensitive) (internal sequence) *WB detects various isoforms	WB, IHC-P, IP
Abcam	ab187041	Rabbit monoclonal [EPR17805-146] TrkB	Mouse, Rat	Recombinant fragment within Mouse TrkB aa 100-450. The exact sequence is proprietary.	WB, IP, IHC-P
Abcam	ab6180	Rabbit	Rat, Human	Synthetic peptide corresponding to Rat TrkB aa 23-36 (extracellular) conjugated to Keyhole Limpet Haemocyanin (KLH) (Glutaraldehyde). Sequence: AFPRLEPNSIDPENC	IHC-P, ICC/IF, IHC-Fr
Abcam	ab89925	Mouse monoclonal [MM0586-7Y6] TrkB	Human	Human TrkB recombinant protein	WB
Abcam	ab43079	Rabbit	Mouse, Rat	Extracellular domain of Mouse TrkB.	ELISA, IHC-Fr
Abnova	H00004915-M02	Mouse	Human	NTRK2 (AAH31835, 1 a.a. ~ 477 a.a) full-length recombinant protein with GST tag.	WB, ELISA
Cell Signaling	4607	Rabbit TrkB (80G2)	Human, 100% predicted with Mouse, Rat	Synthetic peptide surrounding Pro50 of human TrkB.	IHC, F
Cell Signaling	4603	Rabbit TrkB (80E3)	Human, Rat, Mouse	Synthetic peptide surrounding Pro50 of human TrkB.	WB
Cell Signaling	4606	Rabbit	Human	Synthetic peptide corresponding to human TrkN	WB
EMD Millipore	AB9872	Rabbit	Mouse, rat	Extracellular domain of recombinant mouse receptor expressed in mammalian cells	IHC
EMD Millipore	AB07-225	Rabbit	Mouse, rat Immunizing sequence has 97% identity with mouse TrkB and 88% identity with human TrkB.	The entire extracellular domain (corresponding to residues 1–429) of the rat TrkB receptor, expressed in COS cells.	WB
EMD Millipore	AB5372	Rabbit	Human, rat	Amino acids 54-67 of rtrkB	WB, IHC

Supplementary Table 1. Commercially available TrkB antibodies.

R&D	MAB397	Mouse monoclonal IgG_{2B} Clone # 75133	Human	Mouse myeloma cell line NS0-derived recombinant human TrkB Cys32-His430	ELISA, WB
R&D	AF1494	Goat	Mouse	Mouse myeloma cell line NS0-derived recombinant mouse TrkB Cys32-His429	WB, ELISA
R&D	MAB3971	Mouse monoclonal IgG_1 Clone # 72509	Human	Mouse myeloma cell line NS0-derived recombinant human TrkB Cys32-His430	WB, ELISA
R&D	AF397	Goat	Human	Mouse myeloma cell line NS0-derived recombinant human TrkB	WB, ELISA
Santa Cruz	sc-8316	Rabbit polyclonal IgG TrkB (H-181)	Recommended for detection of Trk B splice variants L1 and L10 of mouse origin, Trk B gp95 and Trk B gp145 of rat origin and Trk B, Trk B-T1 and Trk B T-Shc of human origin by WB, IP, IF, IHC(P) and ELISA; also reactive with additional species, including and equine, canine, bovine and porcine	Epitope corresponding to amino acids 160-340 mapping within the extracellular domain of Trk B of human origin	IHC-P, WB, IP, IF, ELISA
Santa Cruz	sc-12	Rabbit polyclonal IgG TrkB (794)	Recommended for detection of the Trk B splice variants L1 and L10 of mouse origin, Trk B gp145 of rat origin and Trk B of human origin; also reactive with additional species, including and equine, canine and porcine	Epitope mapping within a C-terminal cytoplasmic domain of Trk B of mouse origin	WB, IP, IF, IHC-P and ELISA
Santa Cruz	sc-20542	Goat polyclonal IgG Trk B (N-20)	Recommended for detection of Trk B splice variants L1 and L10 of mouse origin, Trk B gp95 and Trk B gp145 of rat origin and Trk B, Trk B-T1 and Trk B T-Shc of human origin by; also reactive with additional species, including canine	Epitope mapping within an extracellular domain of Trk B of human origin	WB, IP, IF and ELISA
Santa Cruz	sc-7268	Mouse monoclonal IgG_{2a} Kappa light chain Trk (B-3)	Recommended for detection of Trk A, Trk B and Trk C of mouse, rat and human origin; also reactive with additional species, including and bovine and porcine	Specific for an epitope mapping between amino acids 783-796 at the C-terminus of Trk of human origin	WB, IP, IF, IHC-P and ELISA;
Santa Cruz*	sc-119	Rabbit polyclonal IgG TrkB (C-13)	Recommended for detection of Trk B splice variants L1 and L10 of mouse origin, Trk B gp95 of mouse and rat origin and Trk B-T1 of human origin, also reactive with additional species, including and equine, canine, bovine, porcine and avian	Epitope mapping at the C-terminus of TrkB of mouse origin	WB, IP, IF and ELISA;
Santa Cruz	sc-377218	TrkB Antibody (F-1) is a mouse monoclonal mouse IgM κ (kappa light chain)	Recommended for detection of TrkB, TrkB-T1 and TrkBT-Shc of human origin; Trk B splice variants L1 and L10 of mouse origin; and Trk B gp95 and Trk B gp145 of rat origin	Specific for an epitope mapping between amino acids 37-75 within an extracellular domain of Trk B of human origin	WB, IP, IF, IHC-P and ELISA

Supplementary Table 1. Commercially available TrkB antibodies.

Santa Cruz	sc-139	Rabbit polyclonal IgG Trk Antibody (C-15)	Recommended for detection of Trk A, Trk B and Trk C of mouse, rat, human and avian origin; also reactive with additional species, including and equine, canine, bovine, porcine and avian	Epitope mapping at the C-terminus of Trk of porcine origin	WB, IP, IF, IHC-P and ELISA
Santa Cruz	sc-136991	Mouse Monoclonal IgG_1 (kappa light chain) TrkB (G-11)	Human	Raised against amino acids 209-298 of Trk B of human origin	WB, IP, IF and ELISA
ThermoFisher/ Invitrogen	OST00118G	Sheep	Mouse, Rat	Synthetic peptide corresponding to extracellular domain of Mouse TrkB.	WB, IHC
ThermoFisher/ Invitrogen	PA1-24831	Goat	Human	Recombinant, full length human protein expressed in NSO cells.	ELISA, WB
ThermoFisher/ Invitrogen	PA1-18402	Rabbit	Human, Rat	Synthetic peptide corresponding to residues A(23) F P R L E P N S I D P E N C(36) of rat TrkB.	IHC
ThermoFisher/ Invitrogen	PA1-18403	Rabbit	Mouse, Rat	Extracellular domain of glycosylated mouse TrkB protein produced in CHO cells.	IHC, ELISA
ThermoFisher/ Invitrogen	PA5-34026	Rabbit	Percent identity with other species by BLAST analysis: Human, Chimpanzee, Gorilla, Orangutan, Gibbon, Monkey, Marmoset, Panda, Horse, Rabbit (100%) Bovine (92%).	Synthetic 12 amino acid peptide from cytoplasmic domain of human NTRK2 / TRKB	IHC-P
ThermoFisher/ Invitrogen	MA5-14903	Rabbit	Human, Mouse, Rat	Synthetic peptide surrounding Pro50 of human TrkB	WB
ThermoFisher/ Invitrogen	OST00125W	Rabbit	Human	Synthetic peptide from extracellular domain of rat TrkB	WB, IHC
Origene	TA500386	Mouse Clone OTI2E1 (formerly 2E1)	Human	Full-length protein expressed in 293T cell transfected with human NTRK2 expression vector	WB, IHC, IF
Origene	TA325757	Rabbit	Human, Mouse Rat	Peptide derived from human Trk B	WB, IHC

*Santa Cruz sc-119 only antibody designed with immunogen TrkB.T1 variant (proprietary): picks up WB signal in TrkB.$T1^{-/-}$ mice; very recently discontinued by SC with SC providing suggested link for sc-377218 which picks up all variants (ECD designed) as replacement

ELISA: Enzyme-linked immunosorbent assay
F: Flow cytometry
ICC: Immunocytochemistry
IF: Immunofluorescence
IHC: Immunohistochemistry
IHC-P: Immunohistochemistry-Paraffin
IHC-Fr: Immunohistochemistry-Frozen
IP: Immunoprecipitation
WB: Western Blot
