# Supplementary material for: A kinase-deficient NTRK2 splice variant predominates in glioma and amplifies several oncogenic signaling pathways
Source: Nat Commun. 2020 Jun 12;11:2977. doi: 10.1038/s41467-020-16786-5 (PMC7293284; doi:10.1038/s41467-020-16786-5)
Supplement: Supplementary file 3 — Description of Additional Supplementary Files [file 41467_2020_16786_MOESM3_ESM.pdf]

Description of Additional Supplementary Files

File Name: Supplementary Data 1
Description: TCGA and GTEx Data

File Name: Supplementary Data 2
Description: NTRK Transcripts

File Name: Supplementary Data 3
Description: Mouse Neurospheres and Human GSC Lines

File Name: Supplementary Data 4
Description: Differential Gene Correlation Analysis (DGCA)

File Name: Supplementary Data 5
Description: DE Analysis: GSC Lines (448, 559, G15, G179)

File Name: Supplementary Data 6
Description: DE Analysis: NSC RNA Seq
