# Supplementary material for: A kinase-deficient NTRK2 splice variant predominates in glioma and amplifies several oncogenic signaling pathways
Source: Nat Commun. 2020 Jun 12;11:2977. doi: 10.1038/s41467-020-16786-5 (PMC7293284; doi:10.1038/s41467-020-16786-5)
Supplement: Supplementary file 10 — Reporting Summary [file 41467_2020_16786_MOESM10_ESM.pdf]

nature research

Corresponding author(s): Eric C. Holland
NCOMMS-19-34614A

Last updated by author(s): May 15, 2020

Reporting Summary

Nature Research wishes to improve the reproducibility of the work that we publish. This form provides structure for consistency and transparency in reporting. For further information on Nature Research policies, see Authors & Referees and the Editorial Policy Checklist .

Statistics

For all statistical analyses, confirm that the following items are present in the figure legend, table legend, main text, or Methods section.

n/a	Confirmed	
☐	☒	The exact sample size (*n*) for each experimental group/condition, given as a discrete number and unit of measurement
☒	☐	A statement on whether measurements were taken from distinct samples or whether the same sample was measured repeatedly
☐	☒	The statistical test(s) used AND whether they are one- or two-sided<br>*Only common tests should be described solely by name; describe more complex techniques in the Methods section.*
☒	☐	A description of all covariates tested
☒	☐	A description of any assumptions or corrections, such as tests of normality and adjustment for multiple comparisons
☐	☒	A full description of the statistical parameters including central tendency (e.g. means) or other basic estimates (e.g. regression coefficient) AND variation (e.g. standard deviation) or associated estimates of uncertainty (e.g. confidence intervals)
☐	☒	For null hypothesis testing, the test statistic (e.g. *F*, *t*, *r*) with confidence intervals, effect sizes, degrees of freedom and *P* value noted<br>*Give P values as exact values whenever suitable.*
☒	☐	For Bayesian analysis, information on the choice of priors and Markov chain Monte Carlo settings
☒	☐	For hierarchical and complex designs, identification of the appropriate level for tests and full reporting of outcomes
☒	☐	Estimates of effect sizes (e.g. Cohen's *d*, Pearson's *r*), indicating how they were calculated

Our web collection on statistics for biologists contains articles on many of the points above.

Software and code

Policy information about availability of computer code

Data collection	Software used:<br>TopHat (v2.0.10), Bowtie (v1.0.0), SAMtools (v0.1.19) and STAR (v2.7.0) were used for alignment. Counting reads for for gene associations against the UCSC genes was done with HTSeq(v 0.11.0) Transcript quantification was done used Cufflinks (v2.2.173)<br>R version (3.3.2) was used for all analysis. The following R packages were used: edgeR(v3.28.1), ggplot2(v 2.2.1), pheatmap (v 1.0.12), clusterProfiler (v 3.4.4), DGCA(v 1.0.2)
Data analysis	For the Human GSC dataset, RNA-seq samples were analyzed using the nextpresso pipeline as follow: reads were aligned to the human genome (hg19) with TopHat-2.0.10 using Bowtie 1.0.0 and SAMtools 0.1.19, allowing 3 mismatches and 20 multi-hits; transcripts quantification was calculated with Cufflinks 2.2.173, using the human hg19 transcript annotations from https://ccb.jhu.edu/software/tophat/igenomes.shtml.<br><br>For NSC cell lines dataset, RNA-seq reads were aligned to the UCSC hg19 assembly using STAR2 and counted for gene associations against the UCSC genes database with HTSeq. Differential Gene Expression analysis was using the R/Bioconductor package edgeR.<br><br>For principal component analysis (PCA) plots, the R function 'dist' was used to calculate Euclidean distance between samples. We computed the PCA for the above distances and visualized it using R package ggplot2 (v 2.2.1). Transcript Data was visualized using boxplots using R package ggplot2 . Whole gene correlations between Normal Brain Data from GTEX and LGG, GBM data from TCGA was calculated using R package DGCA (https://cran.r-roject.org/web/packages/DGCA/index.html). For predominant transcript analysis, log2 TPM values for each transcript were compared and each sample from pooled normal brain (GTeX), LGG (TCGA) and GBM (TCGA) was marked as either "TrkB.T1" or "TrkB.FL", depending on which transcript, of the two, had the greater value. GO and Reactome Pathway enrichment analysis was done using R Bioconductor Packages

clusterProfiler v 3.4.4 and dot plots were made using R Bioconductor package DOSE . Heatmaps were made using R package pheatmap (https://CRAN.R-project.org/package=pheatmap).

For manuscripts utilizing custom algorithms or software that are central to the research but not yet described in published literature, software must be made available to editors/reviewers. We strongly encourage code deposition in a community repository (e.g. GitHub). See the Nature Research guidelines for submitting code & software for further information.

Data

Policy information about availability of data

All manuscripts must include a data availability statement. This statement should provide the following information, where applicable:

- Accession codes, unique identifiers, or web links for publicly available datasets
- A list of figures that have associated raw data
- A description of any restrictions on data availability

Publicly available whole gene RPKM read counts and Transcript Data for TCGA-LGG and TCGA-GBM were downloaded from UCSC's Xena Browser (https://xenabrowser.net/datapages/?host=https://tcga.xenahubs.net). Whole gene RPKM read counts and Transcript Data for The Genotype-Tissue Expression (GTEx) Project (v6)48 was obtained from https://gtexportal.org/ (version 6).

Raw data files have been deposited to the NCBI Gene Expression Omnibus under accession number GEO: GSE136868(https://www.ncbi.nlm.nih.gov/geo/query/acc.cgi?acc=GSE136868)

No restrictions on data availability.

Field-specific reporting

Please select the one below that is the best fit for your research. If you are not sure, read the appropriate sections before making your selection.

- [x] Life sciences
- [ ] Behavioural & social sciences
- [ ] Ecological, evolutionary & environmental sciences

For a reference copy of the document with all sections, see nature.com/documents/nr-reporting-summary-flat.pdf

Life sciences study design

All studies must disclose on these points even when the disclosure is negative.

Sample size	Sample sizes were chosen based on preliminary data, previous studies of survival using RCAS-PDGFB driven gliomas in Nestin (N)/tv-a(agouti) performed within our laboratory and current standards as described in Charan & Kantharia (2013) J Pharmacol Pharmacother, 4(4):303-306 (in order to generate statistically significant data at $p < 0.05$).
Data exclusions	No data were excluded.
Replication	Cell culture, RNA Seq, and drug studies were repeated in triplicate, per condition, unless noted otherwise in materials and methods. All data from these replicates was used to generate plots and error bars shown main and supplementary figures. Replicates for IHC on mouse and human tumors and western blots were successful and representative images were chosen for main and supplementary display figures.
Randomization	Mice were randomly assigned to either RCAS-PDGFB or RCAS-PDGFB+RCAS-TrkB.T1 groups. For in vitro experiments, the same cells were split into multiple plates prior to lentiviral infection to eliminate any potential confounds of using different batches of pre-transduced cells.
Blinding	Researchers were not blinded to treatment conditions as all cells or mouse cages were clearly labeled with RCAS or lentiviral vectors used. As cells and mice were often transported to various tissue culture hoods, incubators, or rooms, clear labels ensured that researchers would not mix up cells or mice.

Reporting for specific materials, systems and methods

We require information from authors about some types of materials, experimental systems and methods used in many studies. Here, indicate whether each material, system or method listed is relevant to your study. If you are not sure if a list item applies to your research, read the appropriate section before selecting a response.

Materials & experimental systems

n/a	Involved in the study
☐	☒ Antibodies
☐	☒ Eukaryotic cell lines
☒	☐ Palaeontology
☐	☒ Animals and other organisms
☒	☐ Human research participants
☒	☐ Clinical data

Methods

n/a	Involved in the study
☒	☐ ChIP-seq
☒	☐ Flow cytometry
☒	☐ MRI-based neuroimaging

Antibodies

Antibodies used

Antibodies
For TrkB.T1 immunohistochemistry, the generated mouse TrkB.T1 antibody was used (1.3mg/ml) at 1:20 and recombinant rabbit fusion SPEH1_D12 (11.5mg/ml) was used at 1:500 and TrkB kinase antibody (abcam #ab18987; lot: GR3280550-2) at 1:250. For western blot analyses of human brain, mouse brain, and 3T3-Tv-a cells, commercial antibodies were used according to manufacturer specifications and bands were confirmed by size using Spectra™ Multicolor Broad Range Protein Ladder (ThermoScientific™ catalog # 26634; lot: 00784968): pSTAT3 (Tyr705, Cell Signaling #9145; lot: 22 at 1:1000), pAkt (Ser473, Cell Signaling #4060; lot: 23 at 1:1000), pERK (Cell Signaling phospho-p44/42 MAPK (ERK1/2) (Thr202/Tyr204) (D13.14.4E) XP® #4370; lot: 12 at 1:2000), β-actin (Sigma #AB1978 Clone AC-15; lot: 021M4821 at 1:10,000), pS6 ribosomal protein (Ser235/236, Cell Signaling #4858; lot: 16 at 1:1000), TrkB (Millipore #07-225; lot: 2187222 and lot: 3277578 @ 1:3000), TrkB (abcam #ab33655; lot: GR266297-1 at 1:1000), TrkB (Abcam #ab18987; lot: GR3280550-2 at 1:2000), pPDGFR β (Y1021; Abcam #ab62437; lot: GR38791-13 at 1:200), PDGFR β (Cell Signaling #3169; lot: 13 at 1:800), ERBB3 (Cell Signaling #12708; lot: 4 at 1:1000), NRG2 (Abcam #ab220615; lot: GR3181158-4 at 1:200), vinculin (Sigma-Aldrich, Cat. V9131; lot: 118M4777V at 1:10.000) and added in 5% BSA/TBST overnight at 4 degrees C.

Validation

Antibodies were used according to manufacturer specifications and titrated or altered in an assay dependent fashion. All bands observed in western blots were confirmed by size using Spectra™ Multicolor Broad Range Protein Ladder (ThermoScientific™ catalog # 26634; lot: 00784968). For immunohistochemistry, positive control and negative control slides were used for each individual run on the Ventana platform for validation of staining. Validation of the TrkB.T1 antibody generated for this manuscript is described in the methods section and in Supplementary Data.

Eukaryotic cell lines

Policy information about cell lines

Cell line source(s)

NSC and GSC lines are primary patient derived cells (isolated and maintained in Squatrito (CNIO) and Paddison (Fred Hutchinson Cancer Research Center) labs, mouse tumorspheres are from primary mouse tumors (Squatrito lab, CNIO), and mouse neurospheres derived from primary mouse neural stem cells (harvested at postnatal day 1) (Holland lab, Fred Hutchinson Cancer Research Center). DF1 cells and 3T3 cells stably expressing tva receptor were established in the Holland lab (Ozawa et al (2014) Cancer Cell, 26:288-300) and used for RCAS experiments. 293T cells were maintained in the Paddison lab, used in the Holland lab for lentiviral production for NSC experiments.

Authentication

Authentication was not performed on primary patient derived cells or tumorspheres from primary mouse tumors.

Mycoplasma contamination

Cells were routinely tested for mycoplasma contamination via PCR and results confirmed that there was no contamination.

Commonly misidentified lines
(See ICLAC register)

There were no commonly misidentified lines used in this manuscript.

Animals and other organisms

Policy information about studies involving animals; ARRIVE guidelines recommended for reporting animal research

Laboratory animals

Nestin (N)/tv-a(agouti) pups (P0-P1; male and female) were injected intracranially with either RCAS-PDGFB or RCAS-TrkB.T1 + RCAS-PDGFB expressing DF-1 cells, as described in Materials and Methods, and monitored daily for tumor related symptoms for the duration of the experiment. Upon weaning (~P21), mice were housed with same-sex littermates, with no more than 5 per cage and given access to food/water ad libitum. For antibody generation (as described in materials and methods), adult (7 weeks and older) TrkB.T1-null mice (Dorsey et al. (2006) Neuron (51):21-28 ; a generous gift from Francis S. Lee at Weill Cornell Medical College; equal numbers male and female) were also housed with same-sex littermates, no more than 5 per cage with access to food/water ad libitum.

Wild animals

No wild animals were used in this study.

Field-collected samples

No field collected samples were used in this study.

Ethics oversight	All animal experiments were approved by and conducted in accordance with the Institutional Animal Care and Use Committee of Fred Hutchinson Cancer Research Center (protocol #50842).

Note that full information on the approval of the study protocol must also be provided in the manuscript.
